# The Effect of Correction Algorithms on Knee Kinematics and Kinetics during Gait of Patients with Knee Osteoarthritis

**DOI:** 10.1155/2020/8854124

**Published:** 2020-11-23

**Authors:** Hanna Ulbricht, Meijin Hou, Xiangbin Wang, Jian He, Yanxin Zhang

**Affiliations:** ^1^Human Movement Science, Chemnitz University of Technology, Chemnitz 09112, Germany; ^2^Department of Exercise Sciences, University of Auckland, Auckland 1142, New Zealand; ^3^National-Local Joint Engineering Research Center of Rehabilitation Medicine Technology, Fujian University of Traditional Chinese Medicine, Fuzhou 350122, China; ^4^Key Laboratory of Orthopedics & Traumatology of Traditional Chinese Medicine and Rehabilitation (Fujian University of Traditional Chinese Medicine), Ministry of Education, Fuzhou 350122, China; ^5^College of Rehabilitation Medicine, Fujian University of Traditional Chinese Medicine, Fuzhou 350122, China

## Abstract

In gait analysis, the accuracy of knee joint angles and moments is critical for clinical decision-making. The purpose of this study was to determine the efficacy of two existing algorithms for knee joint axis correction under pathological conditions. Gait data from 20 healthy participants and 20 patients with knee osteoarthritis (OA) were collected using a motion capture system. An algorithm based on Principal Component Analysis (PCA) and a functional joint-based algorithm (FJA) were used to define the knee joint flexion axis. The results show that PCA decreased crosstalk for both groups, and FJA reduced crosstalk in patients with knee OA only. PCA decreased the range of motions of patients with knee OA in the direction of abduction/adduction significantly. There was a significant increase in the maximum knee flexion moment of patients with knee OA by FJA. The results indicate that both algorithms can efficiently reduce crosstalk for gait from patients with knee OA, which can further influence the results of knee joint angles and moments. We recommend that the correction algorithms be applied in clinical gait analysis with patients with knee OA.

## 1. Introduction

In the past few decades, clinical gait analysis has been widely used to assess movement quality and the effect of therapeutic interventions for patients with knee osteoarthritis (OA). In gait analysis, the positions of anatomical landmarks are usually determined by markers placed on the skin. For this group, common symptoms like the joint space narrowing and bone spurs can affect the morphology of the knee, which makes it more challenging to determine the bony marker positions. There are two common problems affecting measurement error, which are the inaccuracy of the marker placement and the soft tissue artifact by the skin-attached marker sets [1]. Since each segment coordinate system is orientated based on the positions of the markers, small changes of these positions can lead to inaccuracy of the axes and errors in angle curves. For example, if the knee joint flexion axis, normally determined by the medial and lateral epicondyle markers, is misaligned, this will make the joint coordinate system not aligned with axes about which rotations are assumed to occur [2]. This misalignment of the knee joint coordinate system will subsequently cause changes of the knee varus-valgus and axial rotation angles. This phenomenon is called crosstalk [3].

Numerous methods have been proposed for accurately defining the knee joint flexion axis. Earlier methods [4–8] are based on knee alignment device, functional axis, and different optimization algorithms. More recently, algorithms based on Principal Component Analysis (PCA) have been used as post hoc procedures to reduce crosstalk [3, 9], which use the transformation of the joint axes to determine the directions with linearly uncorrelated variables. While these methods were found to reduce both the variability associated with repeated hip axial rotation measurements and the knee joint angle crosstalk, these were mainly demonstrated using gait data for healthy participants [9]. In comparison with healthy participants, patients with pathological gait may have bone deformation, abnormal joint malalignments, and contracture, which make it more difficult to determine the landmark positions.

Therefore, subsequent work is needed to compare the effectiveness of the current algorithms under various challenging conditions of pathological knees [9]. In addition, as the crosstalk affects the orientations of the joint axes, it may further change the values of joint moments, which are decomposed based on the joint axes. The effect should be investigated as joint moments are important parameters for clinical gait analysis. For patients with knee OA, previous research showed that the knee adduction moment (KAM) was correlated to cartilage thickness (normalized by the medial to lateral ratio) [10]. Another study showed that there was a significant increase in KAM for patients at later stages of knee OA [11]. It was also reported that the increase of medial contact force (measured by a force-measuring knee implant) could be explained by the increase of the peak value knee flexion moment (KFM) [12]. Therefore, the KFM and KAM have been used as indicators of increased dynamic loads on the medial compartment of the knee and an indicator of the development or progression of OA, respectively [13–17]. However, the effect of crosstalk on joint moments has never been quantified, and the clinical influence is still unclear.

Therefore, this research was aimed at evaluating the efficacy of two established algorithms on gait data from patients with knee OA. An algorithm based on Principal Component Analysis (PCA) [9] and a functional joint-based algorithm (FJA) [18] were used to define the knee joint flexion axis. Our primary hypothesis was that both algorithms would reduce crosstalk in patients with knee OA. Furthermore, we hypothesized that the correction algorithms could significantly increase peak KFM and reduce peak KAM. Systematically testing knee axis correction algorithms on knee OA patients is an important step in determining the reliability and efficacy of these algorithms in clinical practice. Although the study is focused on knee OA patients, patients with bone deformities could be also analysed.

## 2. Materials and Methods

### 2.1. Participants

Twenty healthy adults (age 43.9 ± 19.7 years, weight 59.13 ± 7.49 kg, and BMI 21.7 ± 1.6 kg/m^2^) and 20 patients with knee OA (age 65.7 ± 8.8 years, weight 58.85 ± 6.04 kg, and BMI 23.6 ± 2.5 kg/m^2^) were recruited from the community and the outpatient department of the Affiliated Rehabilitation Hospital of Fujian University of Traditional Chinese Medicine. Participants were recruited via posters on community bulletin boards, mailed leaflets, and advertisements at the hospital recruiting station with ethical approval. Clinical diagnosis of bilateral knee OA was confirmed in accordance with the 2010 Clinical Diagnostic Criteria for the Diagnosis and Treatment of Osteoarthritis by the Chinese Medical Association Rheumatology Branch [19]. Specifically, the diagnosis was confirmed if items1 + 2 + 3 + 4, items1 + 2 + 5, or items1 + 4 + 5matched (item 1: knee pain most of the time in the past month; item 2: crepitus; item 3: morning stiffness lasting ≤30 min; item 4: age ≥ 38 years; and item 5: palpable bony enlargement).

Inclusion criteria of the healthy group are as follows: no knee pain during the previous six months during daily activities, no neurological conditions, and no other visual, auditory, or cognitive impairments that affected walking. All participants signed an informed consent document approved by the Institutional Review Board.

### 2.2. Data Collection and Processing

**Figure 1:**
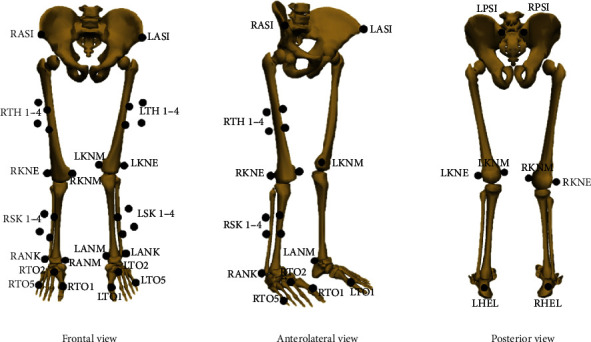
Illustration of the marker set.

Three-dimensional movement data were collected by a ten-camera Qualisys Motion Capture System (Qualisys Track Manager, Sweden) with a sampling frequency of 100 Hz. Ground reaction forces were synchronously collected by two force plates (AMTI 400600 HF-2000, Advanced Mechanical Technology Inc., USA) with a sampling frequency of 2000 Hz. Two experienced lab technicians placed retroreflective markers on the lower body of participants. Markers were placed on the pelvis, feet, thigh clusters, lateral and medial epicondyles of the knee, shank clusters, and lateral and medial malleoli of the ankle (Figure 1).

Before every test session, each participant performed a static trial, in which the participant stood still for a few seconds. This allowed the medial and lateral markers placed on the knee and ankle to be determined by the triad markers on the shank and thigh through the calibrated anatomical system technique [20]. The knee and ankle joint markers were removed after the static trial. Participants were then required to perform five walking trials barefoot on the floor at a self-selected speed. A fourth-order Butterworth low-pass filter was used to filter the marker trajectories with a cut-off frequency of 6 Hz. Anatomical coordinate systems were defined for the pelvis, thigh, shank, and foot segments following the International Society of Biomechanics recommendation [21, 22]. After the segment coordinate systems were defined, the orientations of the referred segment with respect to its reference segment were represented by a sequence of three rotations following the flexion/extension, adduction/abduction, and internal/external order [23]. An inverse dynamic analysis procedure was used to calculate the joint angles and joint moments [24]. The calculation of the kinematic and kinetic data was conducted using Visual 3D Version 6 (C-Motion Inc., Germantown, MA, USA).

### 2.3. Correction Procedure

Two different algorithms, FJA and PCA, were used for data correction. The FJA is a method for calculating functional joint axes and joint centers developed by Schwartz and Rozumalski [18], in which the joint can be estimated as the point on the segment that moves least in relation to the other segment. The results showed that the method is an objective and precise approach [18].

The PCA algorithm is a post hoc procedure that uses the transformation of the axes to identify the direction of the greatest variance (principal component), which is used to calculate the corrected knee joint axes [9]. By mathematically formulating a quadratic optimization problem, this algorithm could reduce both the variability associated with repeated rotation measurements and the joint angle crosstalk.

Both algorithms were implemented using the Visual 3D software platform with the following steps: (1) A technical coordinate system was set up based on the thigh and shank cluster markers to represent each segment for the computations. (2) The data from a walking trial were used as the input to run the algorithm. After implementation, two additional landmarks (the lateral femoral epicondyle and the knee joint center) were created, and the mediolateral knee axis was determined by the landmarkers. (3) Thigh segment coordinate systems were refined based on the markers' position. That is, the original marker-based coordinate system was modified as the PCA-corrected coordinate system or the FJA-corrected coordinate system. The knee flexion axis is defined in the plane of the knee joint center, hip joint center, and lateral epicondyle and in the direction perpendicular to the long axis of the thigh. (4) An inverse dynamic analysis procedure was used to calculate the joint angles and joint moments.

Previous studies quantified crosstalk by using the correlation and changes in the range of motion (ROM) profile of abduction/adduction and flexion/extension angle [3, 4]. Similarly, we calculated the coefficient of determination between knee adduction and flexion throughout the gait cycle (*R*^2^), ROM, and the offset and variability of abduction/adduction and axial rotation angles to evaluate the efficacy of the algorithms in the reduction of crosstalk. In addition, the joint moments were also calculated to test if the correction of the knee axis will affect the moment calculation.

### 2.4. Statistical Analysis

One out of the five collected trials was used to run the algorithm to determine the landmarks at the knee joint. Three out of the left four trials were processed to calculate the kinematic and kinetic variables. Parameters of all three walking trials were averaged for all participants. For all parameters, the mean differences between corrected and uncorrected data are presented with standard deviations. To determine the level of effect that the correction algorithms have, a one-way analysis of variance (ANOVA) with repeated measure was conducted on the kinematic and kinetic parameters, and the partial eta squared (*η*^2^) was calculated as a measure of effect size. The effect size was considered as small (*η*^2^ = 0.01), moderate (*η*^2^ = 0.06), or large (*η*^2^ = 0.14) [25]. When significant results were found, post hoc analysis was made with Bonferroni pairwise comparison. Statistical analysis was conducted using IBM SPSS 24 (SPSS Inc., Chicago, USA). Differences were considered to be significant for *p* < 0.05.

## 3. Results

PCA significantly decreased *R*^2^ in both healthy participants (*p* = 0.03) and patients with knee OA (*p* < 0.001). In comparison to the FJA, PCA performance was superior in healthy participants. Whilst there was a significant decrease in *R*^2^ by PCA, no such effect was found by FJA (Table 1). However, for the patients with knee OA, both algorithms decreased correlation significantly (PCA: *p* < 0.001; FJA: *p* = 0.009). Although the PCA reduced crosstalk a bit more than FJA, no significant difference was found between both algorithms. The resulting partial eta-squared value indicates a large effect for patients with knee OA (*η*^2^ = 0.581) and for healthy subjects (*η*^2^ = 0.394) according to Cohen [25].

The PCA corrected the knee abduction/adduction ROM for patients with knee OA (*p* = 0.016); however, there was no significant influence on mean offset or any other knee kinematics (Table 1). PCA significantly reduced abduction/adduction ROM of patients with knee OA by 2.6 degrees (*p* = 0.016), whilst there was no such effect by FJA. There was a small effect on abduction/adduction offset for healthy subjects (*η*^2^ = 0.043) and patients with knee OA (*η*^2^ = 0.02). A moderate effect was found on axial rotation offset for healthy subjects (*η*^2^ = 0.096) and a large effect on patients with knee OA (*η*^2^ = 0.159). However, the abduction/adduction and axial rotation offset did not change significantly.

Changes of the within-limb variability of knee abduction/adduction and axial rotation offset did not significantly differ before and after applying correction algorithms. Partial eta squared indicates moderate effect size on variability of abduction/adduction offset for healthy subjects (*η*^2^ = 0.074) and patients with knee OA (*η*^2^ = 0.073). The variability of axial rotation offset was higher than the variability of the abduction/adduction offset, and the effects were larger with a moderate effect size for healthy subjects (*η*^2^ = 0.118) and a large effect size for patients with knee OA (*η*^2^ = 0.232).

**Figure 2:**
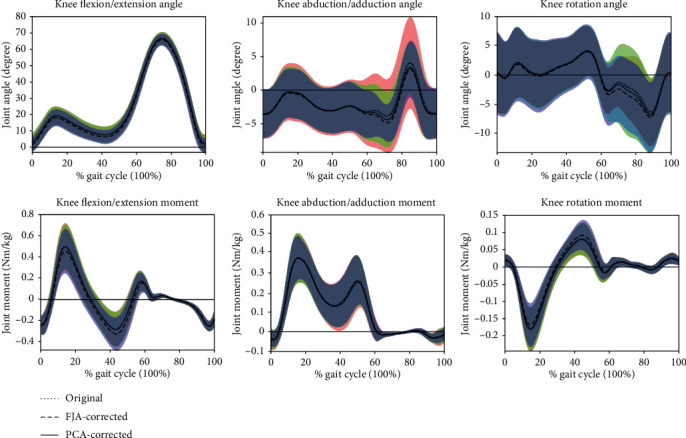
The knee angles and moments of healthy participants during a gait cycle. The shaded areas represent the standard deviation for the original data.

**Figure 3:**
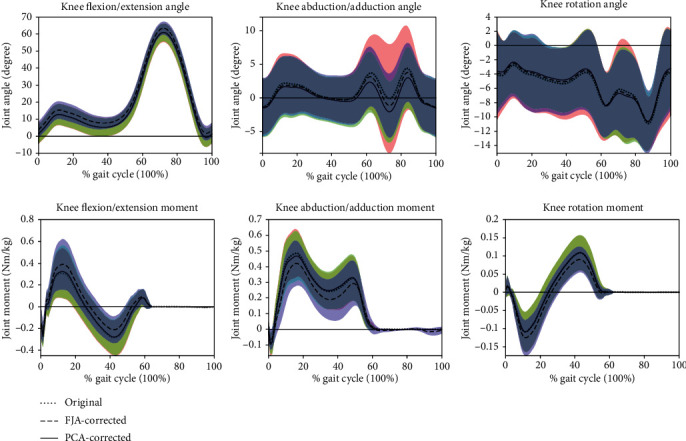
The knee angles and moments of patients with knee OA during a gait cycle. The shaded areas represent the standard deviation for the original data.

There was also a significantly increased maximum KFM of patients with knee OA by FJA from 0.352 to 0.414 Nm/kg (*p* = 0.032). Whilst there is a large effect size on KFM for health subjects (*η*^2^ = 0.156) and patients with knee OA (*η*^2^ = 0.29), as well as KAM of patients with knee OA (*η*^2^ = 0.23), there is no effect on KAM of healthy subjects (*η*^2^ = 0.007). Normalized knee angles and moments are shown in Figures 2 and 3.

## 4. Discussion

The aim of this study was to investigate the ability of two existing compensation algorithms to correct the knee flexion axis under challenging conditions of a pathological knee. To evaluate the efficiency of these algorithms, *R*^2^, ROM, offset variability, and joint moments were calculated. Both algorithms could reduce the correlation of flexion/extension and abduction/adduction for patients with knee OA, suggesting that the compensation algorithms performed well under challenging conditions of pathological knees. The partial eta squared value indicates a moderate to large effect size for most of the parameters. Our results agree with the results from previous studies [9], which showed that the PCA performed well in the healthy group and no effect was found for mean knee offset and offset variability for the standard marker placement.

Previous studies also showed that the abduction/adduction angle becomes increasingly more sensitive to change in the flexion/extension angle with increased misalignment of the flexion axis [26]. Furthermore, a correctly defined flexion axis should decrease the abduction angle and increase the flexion angle [27]. For example, Schwartz and Rozumalski showed significantly reduced abduction/adduction ROM and increased flexion/extension ROM after applying the FJA, indicating improved accuracy [18]. Baudet et al. showed results similar to those from Schwartz and Rozumalski after applying a PCA-based algorithm [3]. In our study, only the abduction/adduction ROM of patients with knee OA reached statistical significance.

Different from the study of Schwartz and Rozumalski [18], we utilized walking trials instead of functional trials for calculating the functional joint. This might be the reason for the lack of significant improvement in ROM in our study. Previous studies used different movements for determining the functional knee axis, and there is no standard for ROM, the kind of movement, and the number of trials yet. Begon et al. investigated the effects of the movement utilized for estimating the hip joint center [28]. They recommend ten cycles of limited movement in the direction of flexion/extension, abduction/adduction, and axial rotation [28]. Although a functional movement is supposed to be less susceptible to human error, using a walking trial for axis calculation is more practical in clinical set-ups, where the time and movement abilities of patients are limited. Further studies are needed for direct comparison of results when using walking trials and functional movement with different algorithms and then to investigate the reliability and reproducibility of those different algorithms in the clinical gait analysis.

Our finding has some clinical implications. OA is a severe disease which affects more than 250 million people around the world. Early identification and monitoring of the conditions can improve treatment outcomes and reduce medical costs. With the technological advances, gait analysis, radiography (X-ray, MRI), and biomarkers are now widely used towards this solution. However, the outcome measures from different assessment techniques do not always match well. For example, it was reported that the gait analysis results (KAM) were not closely correlated with the radiographic measures of OA severity (K-L grade, joint space narrowing) [29]. One possible reason for this mismatch is the measurement error.

As aforementioned, the high crosstalk for the knee OA group may be due to the bony deformation of these patients, which makes it more challenging to accurately locate the anatomical landmark positions. An ill-defined knee flexion-extension axis from these landmarks will result in mixing the original KAM and original KFM. That is, due to the axis shift, the resultant KAM is a combination of the KAM and KFM from the original (true) coordinate system. Moreover, patients with knee OA were found to have the changed “screw-home” mechanism [30, 31] when compared with a healthy population, which was characterized by decreased extension during the stance phase and decreased flexion during push-off and initial swing phase, as well as posterior tibia translation. Therefore, accurate measurement of knee kinematics can better quantify the possible altered biomechanical mechanism in patients with knee OA.

It has been reported that measurement errors exceeding 5 degrees could lead to a misleading clinical interpretation [32]. From Figure 3, we may also speculate that the knee joint flexion axis was refined by the FJA, which results in an increased knee flexion angle and further leads to an increased KFM and a decreased KAM. In this study, we found that the correction algorithms could efficiently reduce *R*^2^. These algorithms can be implemented with popular clinical data processing software like Visual 3D, which makes them practical to be used by clinicians. Further study based on a gold-standard measure (i.e., MRI) needs to be done to further validate if the refined axis is close to the true flexion axis and can be used to replace the traditional method for moment computation. In summary, although the approaches are not brand new, the study is focused on the application and the analysis performed on OA patients. We recommend that, for clinical gait analysis with knee OA patients, the correction algorithms can be applied.

Some limitations of this study should be considered when interpreting these results. As aforementioned, the crosstalk is the error in knee flexion that would affect the knee varus-valgus and axial rotation angles, not the correlation between knee flexion and knee abduction/adduction. Nevertheless, previous studies used the correlation between the knee flexion and adduction as an indicator of a misidentified knee flexion axis and the subsequent kinematic errors [3, 4, 9]. As there is no gold-standard measure for the true flexion axis, more work needs to be done to further validate if the refined axis is close to the true flexion axis. During gait, the rotations in the frontal and transverse planes are limited in range. Therefore, the algorithms can also be tested using other motor tasks.

## 5. Conclusions

Bony deformation of patients with knee OA may lead to the high crosstalk and make it more challenging to put the landmarks exactly on the anatomical positions. However, the compensation algorithms, PCA and FJA, could reduce the correlation of flexion/extension and abduction/adduction for patients with knee OA. So, we recommend that the correction algorithms can be applied in the clinical gait analysis with patients with knee OA.

## Figures and Tables

**Table 1:** Descriptive statistics for the key kinetic and kinematic variables.

		Original mean (SD)	PCA mean (SD)	FJA mean (SD)	Effect size (partial eta^2^)
*R* ^2^	Healthy	0.288 (0.219)	0.129 (0.167)^∗^	0.188 (0.145)	0.394
	Knee OA	0.437 (0.310)	0.096 (0.083)^∗∗^	0.209 (0.200)^∗^	0.581
Knee ROM flex/ext (°)	Healthy	66.034 (4.557)	66.186 (4.638)	66.667 (4.224)	0.171
	Knee OA	62.288 (4.508)	62.526 (4.456)	62.481 (4.458)	0.310
Knee ROM abd/add (°)	Healthy	12.185 (4.503)	10.969 (3.300)	11.468 (3.273)	0.388
	Knee OA	10.798 (3.155)	8.192 (2.901)^∗^	8.960 (2.988)	0.430
Knee ROM axial rotation (°)	Healthy	15.083 (4.813)	15.950 (5.209)	15.880 (4.381)	0.132
	Knee OA	15.328 (4.160)	15.078 (3.160)	14.454 (3.034)	0.305
Knee abd/add offset (°)	Healthy	-1.827 (4.005)	-2.011 (3.243)	-2.171 (2.968)	0.043
	Knee OA	0.509 (3.518)	0.319 (3.928)	0.188 (3.260)	0.020
Variability of knee abd/add offset (°)	Healthy	0.248 (0.141)	0.250 (0.129)	0.242 (0.140)	0.074
	Knee OA	0.248 (0.133)	0.277 (0.123)	0.266 (0.133)	0.073
Knee axial rotation offset (°)	Healthy	-0.216 (4.103)	-0.404 (4.736)	-0.682 (4.779)	0.096
	Knee OA	-5.772 (3.985)	-5.319 (3.799)	-5.637 (3.933)	0.159
Variability of knee axial rotation offset (°)	Healthy	0.681 (0.377)	0.676 (0.370)	0.684 (0.376)	0.118
	Knee OA	0.611 (0.342)	0.633 (0.353)	0.603 (0.338)	0.232
Max KFM (Nm/kg)	Healthy	0.516 (0.204)	0.518 (0.187)	0.477 (0.190)	0.156
	Knee OA	0.352 (0.184)	0.377 (0.138)	0.414 (0.216)^∗^	0.290
Max KAM (Nm/kg)	Healthy	0.408 (0.121)	0.406 (0.119)	0.404 (0.110)	0.007
	Knee OA	0.508 (0.145)	0.473 (0.131)	0.487 (0.156)	0.230

Significant difference between original and corrected data. ^∗^*p* < 0.05; ^∗∗^*p* < 0.001.

## Data Availability

The data that support the findings of this study are available on request from the corresponding author, Yanxin Zhang. The data are not publicly available yet due to the underdevelopment of the project.
